# A Case of Systemic Lupus Erythematosus in a Patient Presenting with Libman-Sacks Endocarditis

**DOI:** 10.1155/2021/5573141

**Published:** 2021-08-31

**Authors:** Mariann Al-Jehani, Faisal Al-Husayni, Ahmed Almaqati, Jomanah Shahbaz, Saad Albugami, Wail Alameen

**Affiliations:** ^1^King Saud bin Abdulaziz University for Health Sciences, Jeddah, Saudi Arabia; ^2^King Abdullah International Medical Research Center, Jeddah, Saudi Arabia; ^3^Department of Internal Medicine, National Guard Hospital, Jeddah, Saudi Arabia; ^4^Department of Cardiac Sciences, King Faisal Cardiac Center, National Guard Hospital, Jeddah, Saudi Arabia

## Abstract

**Background:**

Libman-Sacks endocarditis (LSE) is a rare cardiovascular manifestation of systemic lupus erythematosus/antiphospholipid syndrome that is described as a sterile verrucous nonbacterial vegetative lesion. These lesions can cause progressive damage to the heart valves leading to valve surgery. The most common valves to be affected are the aortic and mitral valves. Libman-Sacks endocarditis is associated with malignancies, other systemic diseases like systemic lupus erythematosus (SLE) and antiphospholipid antibody syndrome (APS). The majority of LSE patients are usually asymptomatic. *Case Summary*. We describe a 39-year-old male patient who presented with increasing shortness of breath and pulmonary congestion. He was found to have severe mitral valve regurgitation and mitral stenosis. Transesophageal echocardiogram confirmed the diagnosis of Libman-Sacks endocarditis with thickened mitral valve leaflets with symmetrical mass-like structure causing a restriction in the valve function during both cardiac phases later diagnosed with systemic lupus erythematosus by immunology. The patient was started on diuretics, anticoagulants, angiotensin inhibitors, beta-blockers, and hydroxychloroquine. He underwent successful mechanical mitral valve replacement with a 27 mm St. Jude valve. The mitral valve was found to be grossly thickened with friable tissue and complete amalgamation of the leaflets with subvalvular apparatus. The patient suffered some warfarin adverse effects a year later but did well otherwise.

**Conclusion:**

This case demonstrates that Libman-Sacks endocarditis can be the first manifestation of systemic lupus erythematosus. Early and prompt diagnosis of LSE can prevent and lessen the many side effects associated with thromboembolism. Additionally, addressing the underlying disease is key to successful treatment.

## 1. Introduction

Libman-Sacks endocarditis (LSE) is noninfectious endocarditis that is sometimes referred to as nonbacterial thrombotic endocarditis (NBTE), marantic endocarditis, or verrucous endocarditis. It is characterized by deposition of thrombi mainly on aortic and mitral valves; involvement of other valves is less common. It was first described by Emanuel Libman and Benjamin Sacks in 1924 [1]. LSE was mostly associated with malignancies, for example, patients with pancreatic adenocarcinoma were found to have a higher risk of developing LSE with systemic embolization being the leading cause of morbidity [2]. Additionally, LSE is linked to other systemic diseases like systemic lupus erythematosus (SLE) and antiphospholipid antibody syndrome (APS). LSE is encountered among 10% of patients with SLE; it correlates with the disease activity, duration, anticardiolipin antibodies, and APS manifestations. SLE patients with LSE frequently have evidence of valvular affection and lesion progression as shown by Moyssakis et al. [1].

The majority of LSE patients are usually asymptomatic, while if the patient was symptomatic it is usually due to embolic infarctions either as cerebrovascular or systemic thromboembolism. SLE and APS patients may present with signs and symptoms of their underlying diseases, such as malar rash and recurrent miscarriages.

The prognosis of LSE has not been well defined; it is usually considered poor, especially if those patients develop recurrent thromboembolism [3].

We are describing an adult patient who presented with LSE as the first manifestation of SLE.

## 2. Case Presentation

**Figure 1:**
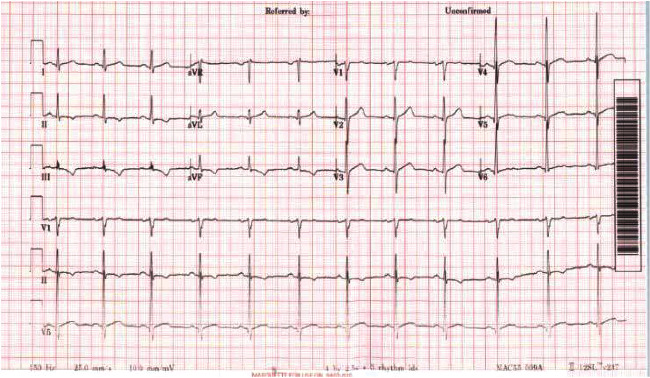
Patient's electrocardiogram showing inverted T-wave in leads II, III, and AVF.

A 39-year-old male, who was medically free prior to this, presented complaining of a three-month history of exertional dyspnea classified as New York Heart Association (NYHA) Class III, associated with cough and palpitation. The patient suffered from five ill-defined syncopal attacks during the past year. He reported a three-month history of lethargy, loss of appetite, and weight loss. The patient denied any personal history of fever, joint pain, or drug abuse. Family history was unremarkable for malignancies or cardiac conditions. Upon admission, the patient was vitally stable with normal saturation on ambient air. Chest examination revealed decreased breath sounds in the right lower zone with bilateral basal crackles, soft first heart sound, normal second heart sound, loud pansystolic murmur, and a soft diastolic rumble at the mitral area. His initial blood work is shown in Table 1. ECG revealed T-wave inversion in leads II, III, and AVF (Figure 1).

**Figure 2:**
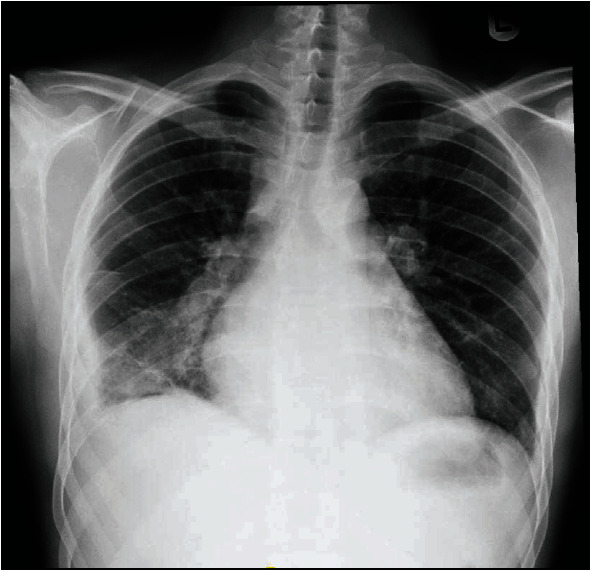
Patient's chest X-ray demonstrating cardiomegaly and right lower lobe consolidations with pleural effusion.

**Figure 3:**
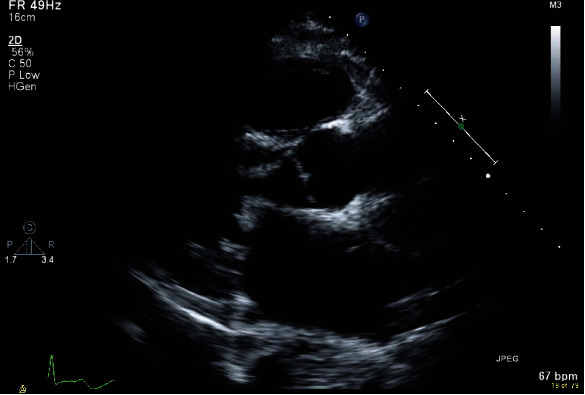
Parasternal long axis view revealing a diffused leaflet thickening of the mitral valve.

**Figure 4:**
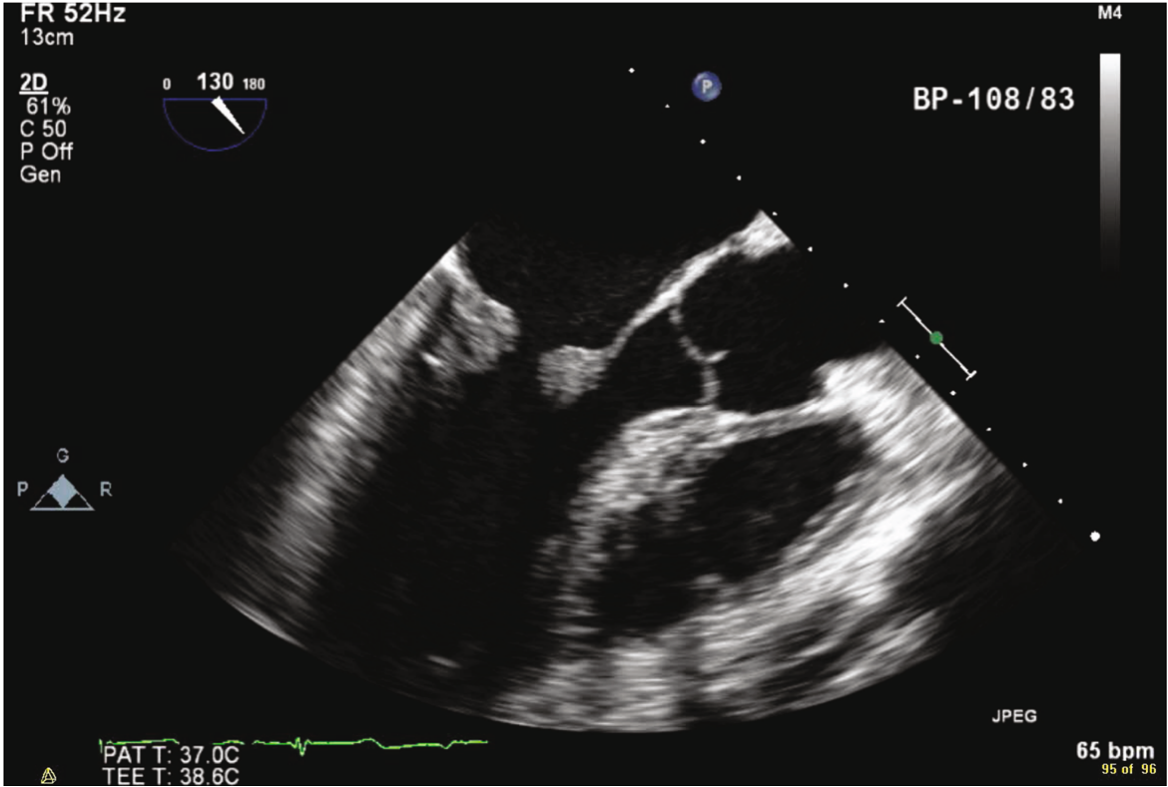
Transesophageal echocardiogram showing a symmetrical mass-like structure on the ventricular surface involving the tips of both leaflets extending to the body, causing a restriction in the valve function.

**Figure 5:**
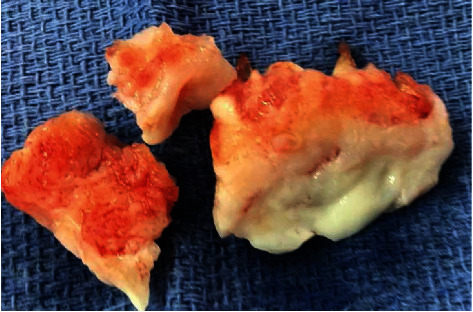
Gross anatomy of the mitral valve leaflets in keeping with Libman-Sacks endocarditis.

Chest imaging revealed cardiomegaly and right pleural effusion with right lower lobe consolidations (Figure 2). Echocardiogram showed a dilated right ventricle with mild to moderate tricuspid regurgitation and severe rheumatic mitral stenosis with moderate to severe mitral regurgitation (Figure 3). The patient was then commenced on diuretics, anticoagulants, angiotensin inhibitors, and beta-blockers. Transesophageal echocardiogram showed thickened mitral valve leaflets with symmetrical mass-like structure on the ventricular surface involving the tips of both leaflets extending to the body, causing a restriction in the valve function during both cardiac phases (Figure 4). Later, the patient underwent a diagnostic angiography, which showed normal coronary arteries. The immunology test came back confirming the diagnosis of SLE (Table 2). Following cardiac team discussion, the patient underwent mitral valve replacement surgery. During the operation, the mitral valve was found to be grossly thickened with friable tissue and complete amalgamation of the leaflets with subvalvular apparatus (Figure 5). The valve was replaced with a 27 mm St. Jude valve. Postop echocardiogram was performed; thus, the surgery was deemed successful and his hospital stay was uneventful. Tissue biopsy showed degenerative changes, myxoid areas with hemorrhage admixed with acute and chronic inflammatory cell infiltrate. In addition, fibroid necrosis was present without calcification. The diagnosis of LSE was confirmed with SLE as underlying disease. The patient was started on hydroxychloroquine 400 mg daily, warfarin, and aspirin. At one-year follow-up, he had an increased intraocular pressure, hyphema, total vitreous hemorrhage, and choroidal hemorrhage. The patient was admitted and underwent hyphema drainage and trabeculectomy. Otherwise, the patient was doing well.

## 3. Discussion

Cardiac involvement in SLE is common. It is estimated that more than 50% of SLE/APS patients have cardiovascular manifestations in the form of pericarditis, myocarditis, LSE, pulmonary arterial hypertension, conduction disease, and coronary artery disease. Left-sided heart valves are by far the most affected. It has previously been reported to have cardiac involvement as the main reason for presentation; however, this is extremely rare [1–8].

Multivalvular involvement often occurs, but typically, the most frequently affected valve is the mitral valve with the vegetations occurring near the edge of the valve or on both of its surfaces. Involvement of the atrial or ventricular endocardium or the chordae tendineae and papillary muscles is rare.

The approach to diagnose LSE might not be simple as some SLE or APS patients are asymptomatic. Echocardiography is the best first modality to diagnose LSE, but transesophageal echocardiography is more sensitive and specific than transthoracic echocardiography [9, 10]. Doppler echocardiography detects between 18% and 50% of valve diseases [1–10], while transesophageal echocardiography detects up to 74% [9, 10]. LSE on imaging is described as irregular heterogeneous echo density with the absence of independent motion of the verrucous vegetations on the cardiac valves and endocardium [9, 10]. The mitral and aortic valves are most affected by leaflet thickening and regurgitation. Although the origin of valvular lesions in SLE is closely linked to the presence of antiphospholipid antibodies [10], negative test results have been described in literature in other patients with SLE and LSE [8] or even in nonbacterial thrombotic endocarditis without underlying disease.

The LSE diagnosis is difficult to be confirmed by laboratory tests; however, patients who are suspected of having LSE must have complete blood count, comprehensive metabolic workup, blood cultures, autoimmune profile, and hypercoagulable workup [1–9].

When treating LSE, the underlying disease, either SLE or APS, must be addressed. Hydroxychloroquine (HCQ), an antimalarial drug, has been an effective option for managing SLE, especially in the early stages [1–10]. Its effectiveness has been established in the mild form of SLE, while not as effective in preventing severe SLE manifestations such as glomerulonephritis and central nervous system involvement [11].

Corticosteroids are considered a treatment option to reduce the inflammatory reaction caused by LSE; however, they can lead to tissue scarring and fibrosis, predisposing to further valvular damage [11, 12]. Anticoagulation should be considered in SLE/APS patients as secondary prevention for thromboembolic events, especially those who have had previous thromboembolic events [11–15].

Surgical valve replacement is recommended for symptomatic and severe cases of LSE. Mechanical valve replacement in females of reproductive age is not preferable as they would require to be put on an anticoagulation regime with increased fetal and maternal side effects. Nonetheless, it is still recommended as many authors believe that SLE/APS patients will eventually end up on anticoagulants for the disease-associated thromboembolism [14, 15].

## 4. Conclusion

LSE is rare as the first manifestation of SLE/APS. We presented a 39-year-old male with LSE as the initial manifestation of SLE. It was successfully diagnosed by echocardiography and treated with mitral valve replacement surgery. The case highlights SLE as a differential diagnosis when encountering a healthy individual with new-onset valve disease. Early and prompt diagnosis of LSE can prevent and lessen the many side effects associated with thromboembolism.

## Figures and Tables

**Table 1:** Initial laboratory data for the patient at presentation.

Labs	Results	Reference range
White blood count	5.5 × 10^9^/L	4.0–11.0 × 10^9^/L
Hemoglobin	11.9 g/dL	11.5–16.5 g/dL
Platelet	111 × 10^9^/L	150–450 × 10^9^/L
Neutrophil count	3.14 × 10^9^/L	2–7.5 × 10^9^/L
Lymphocyte count	1.08 × 10^9^/L	1.5–4 × 10^9^/L
Blood urea nitrogen	8.1 mmol/L	2.1–7.1 mmol/L
Creatinine	150 *μ*mol/L	62–106 *μ*mol/L
Brain natriuretic peptide	1585 pg/mL	<100 pg/mL
High-sensitivity troponin	>0.03 ng/mL	>0.03 ng/mL

**Table 2:** Autoimmune laboratory tests concerning the diagnosis of systemic lupus erythematosus.

Labs	Results	Reference range
Antinuclear antibody	Positive	Negative
Anti-double-stranded DNA	355.7	<68.6 is negative 68.6–229 is moderately positive >229 is strongly positive
Ribonucleoprotein antibody	472.5	<20 is negative 20–39 is weakly positive 40–80 is moderately positive >80 is strongly positive
Anti-Smith antibody	114.71	<20 is negative 20–39 is weakly positive 40–80 is moderately positive >80 is strongly positive
C3 complement	0.8 g/dL	0.9–1.9 g/dL
C4 complement	>0.06	0.1–0.4 g/dL

## Data Availability

The data used to support the findings of this study are included within the article.
